# A novel experimental porcine model to assess the impact of differential pulmonary blood flow on ischemia–reperfusion injury after unilateral lung transplantation

**DOI:** 10.1186/s40635-021-00371-1

**Published:** 2021-02-05

**Authors:** Anna Elisabeth Frick, Michaela Orlitová, Arno Vanstapel, Sofie Ordies, Sandra Claes, Dominique Schols, Tobias Heigl, Janne Kaes, Berta Saez-Gimenez, Robin Vos, Geert M. Verleden, Bart Vanaudenaerde, Stijn E. Verleden, Dirk E. Van Raemdonck, Arne P. Neyrinck

**Affiliations:** 1 Department of Cardiovascular Sciences, KU Leuven, Leuven, Belgium; 2 BREATHE, Department of Chronic Diseases, Metabolism and Ageing (Chrometa), Leuven Lung Transplant Unit, KU Leuven, Leuven, Belgium; 3 Laboratory of Virology and Chemotherapy, Department of Microbiology and Immunology, Rega Institute for Medical Research, KU Leuven, Leuven, Belgium; 4 Lung Transplant Unit, Hospital Universitari Vall D’Hebron, Barcelona, Spain; 5 Department of Respiratory Diseases, University Hospitals Leuven, Leuven, Belgium; 6 Department of Thoracic Surgery, University Hospitals Leuven, Leuven, Belgium; 7 Department of Anesthesiology, University Hospitals Leuven, Leuven, Belgium

**Keywords:** Porcine left lung transplantation, Primary graft dysfunction, Pulmonary vascular resistance

## Abstract

**Background:**

Primary graft dysfunction (PGD) remains a major obstacle after lung transplantation. Ischemia–reperfusion injury is a known contributor to the development of PGD following lung transplantation. We developed a novel approach to assess the impact of increased pulmonary blood flow in a large porcine single-left lung transplantation model.

**Materials:**

Twelve porcine left lung transplants were divided in two groups (*n* = 6, in low- (LF) and high-flow (HF) group). Donor lungs were stored for 24 h on ice, followed by left lung transplantation. In the HF group, recipient animals were observed for 6 h after reperfusion with partially clamping right pulmonary artery to achieve a higher flow (target flow 40–60% of total cardiac output) to the transplanted lung compared to the LF group, where the right pulmonary artery was not clamped.

**Results:**

Survival at 6 h was 100% in both groups. Histological, functional and biological assessment did not significantly differ between both groups during the first 6 h of reperfusion. injury was also present in the right native lung and showed signs compatible with the pathophysiological hallmarks of ischemia–reperfusion injury.

**Conclusions:**

Partial clamping native pulmonary artery in large animal lung transplantation setting to study the impact of low versus high pulmonary flow on the development of ischemia reperfusion is feasible. In our study, differential blood flow had no effect on IRI. However, our findings might impact future studies with extracorporeal devices and represent a specific intra-operative problem during bilateral sequential single-lung transplantation.

## Introduction

Primary graft dysfunction (PGD) occurs within the first 72 h after lung transplantation (LTx) and it is clinically reflected by impaired gas exchange, alveolar infiltrates on chest X-ray, and pulmonary edema representing acute allograft ischemia–reperfusion injury (IRI) [1]. PGD is associated with early morbidity and mortality [2]. PGD has a multifactorial nature with well-studied donor, procedural, and recipient risk factors. The major component responsible for PGD is still ischemia–reperfusion injury (IRI) [3].

The hallmark of IRI is the increased permeability of the alveolo-capillary membrane. Once reperfusion of the transplanted allograft occurs, ROS and pro-inflammatory cytokines activate neutrophils and upregulation of cell-surface adhesion molecules on the endothelial side of the lung occurs. The following disruption of alveolo-capillary membrane results in increased microvascular permeability, increased PVR, impaired oxygenation and eventually pulmonary edema [4, 5]. Endothelial cells are exposed to tangential shear stress and circumferential wall stretch by the blood flow through the pulmonary vasculature [6].

Alterations in endothelial shear stress (such as the interruption and re-installation of flow during IRI) result in a cellular signaling cascade which can contribute to trigger inflammation in the process of IRI itself [7].

Animal models provide a broad study field to verify clinical findings and are the cornerstone of translational research. The single-left porcine LTx model is commonly used to study the early stages of lung transplantation, and especially IRI. The current described single-lung transplant models have some shortcomings. Most models do not clamp the contralateral native lung after the allograft is reperfused. In this way, it is not possible to control the flow, which is an important driver of IRI based on shear stress alterations, over the newly transplanted lung [12–14].

Studies with (partial) clamping of the right PA would therefore help to improve our understanding of IRI. In addition, this might also be important to understand the intra-operative consequences of sequential bilateral lung transplant procedures. During these procedures, the first implanted lung receives the complete cardiac output when the second graft is transplanted. To avoid this overflow to the new lung and to control the pulmonary flow and RV function, installation of extracorporeal techniques is often considered. In this study using healthy pig donor lungs with identical ischemic intervals and lung preservation methods, we wanted to dissect out the impact of pulmonary flow itself during early reperfusion of the allograft in the development of ischemia–reperfusion injury, both to optimize current transplant models and to study the intra-operative clinical issues regarding bilateral sequential lung transplantation without extracorporeal life support (ECLS).

A left-single lung transplantation survival model with clamping of the right pulmonary artery was chosen because sequential bilateral lung transplantation in pigs is not possible because of anatomic differences with the presence of a separate tracheal bronchus to the right upper lobe and an accessory right lower lobe draining into the left inferior pulmonary vein.

We hypothesized that IRI in the allograft is more severe in a high-flow than in a low-flow reperfusion model.

## Materials and methods

This experimental porcine study (topig20 pigs, Zoötechnisch centrum KU Leuven, Lovenjoel, Belgium) was approved by the Ethics Committee on Animal Research KU Leuven (P011/2018). All animals received human care in accordance with “Principles of Laboratory Animal care”, formulated by the National Society for Medical Research and “Guide for the Care and Use of Laboratory Animals”, prepared by the Institute of Laboratory Animal Resources and published by the National Institutes of Health, USA (NIH Publication No. 86–23, revised 1996).

### Study groups

24 domestic male pigs (Topigs 20) were divided into two groups: high-flow (HF) (*n* = 6 × donor + recipient) and low-flow (LF) (*n* = 6 × donor + recipient) group. The mean body weight of the recipient animals was 52.7 (± 0.90) kg in the LF and 52.3 (± 2.07) kg in the HG group. The donor animals had a mean body weight of 50.47 (± 1.19) kg in the LF and 49.5 (± 1.39) kg in the HF group. There was no significant difference in body weight between the groups.

In both groups, lungs were harvested after cold antegrade flush in the donor animal. After 24-h cold ischemia by storage on 4 °C ice, the left graft was transplanted in a recipient animal. 24-h cold storage is a very long period for lung preservation that is not clinically relevant. However, in order to induce sufficient graft injury resulting in IRI, we opted for a model of 24-h cold ischemia reported as a standard model in many other publications investigating IRI.

In the HF group, the right native pulmonary artery was left unclamped for 2 h after reperfusion to avoid imminent right heart failure. Thereafter, the PA was partially clamped for the remaining 4 h to achieve a flow to the transplanted left allograft (target flow 40–60% of total cardiac output). In the LF group, the right PA was not clamped. Hemodynamic parameters and gas exchange were measured during 6 h of reperfusion in both groups.

### Donor procedure

After sedating the donor animal with an intramuscular injection of 5 mg/kg Zoletil 100 (Virbac, Carros, France) and 3 mg/kg Xyl-M 2% (VMD, Arendonk, Belgium), anesthesia was maintained with 10 mg/kg/h propofol, 20 μg/kg/h fentanyl and intermittent boli of pancuronium 2 mg for muscle relaxation. Animals were intubated with a 7.0-mm endotracheal tube and ventilated (Aestiva 3000; GE Healthcare Europe GmbH, Little Chalfont, UK) with a tidal volume (TV) of 8 ml/kg, positive end-expiratory pressure (PEEP) of 5 cmH_2_O and FiO_2_ of 30%. Respiratory rate (RR) was adjusted to end-tidal carbon dioxide levels (ETCO_2_) (45–55 mmHg). A lateral right neck incision was made to access the right carotid artery for invasive monitoring of arterial blood pressure (ABP). Median sternotomy was performed. Prior to cardiac arrest induced by aortic cross-clamping, all animals were anticoagulated with 300 IU/kg heparin. The thymus was resected and the pericardium opened. Inferior (IVC) and superior (SVC) caval veins were isolated, and the aorta was separated before PA cannulation. After ligation of SVC and IVC and aortic cross-clamp, grafts were flushed antegrade via the PA cannula with 2 L (L) of cold (4 °C) buffered OCS® solution (Transmedics, Andover, MA, USA). Heart–lung block was harvested and the trachea was double-clamped with lungs being inflated and maintaining an airway pressure of 15 cmH_2_O. On the back table a retrograde cold flush with 800 mL buffered OCS solution was performed via the pulmonary veins following excision of the heart. Lungs were placed in two plastic bags and stored in OCS® solution at 4 °C for 24 h.

### Recipient procedure

After anesthetizing the recipient animal and maintaining anesthesia as described above for the donor procedure, a central venous catheter was inserted in the internal jugular vein as well as an arterial catheter in the carotid artery. A mini-laparotomy was performed to insert a bladder catheter. Animal body temperature was monitored with a rectal probe. The pig was turned to a right lateral decubitus position and a left thoracotomy in the 4th intercostal space was performed. All animals were heparinized with 300 IU/kg. After dissection of the pulmonary ligament and ligation of the left hemi-azygos vein, a left pneumonectomy was performed. PA pressure (PAP) and left atrium (LA) pressure (LAP) were monitored with catheters inserted in the common PA and LA by direct surgical cannulation. PA blood flow was measured by transonic flowprobes (Transonic Systems Inc.®, Ithaca, NY) based on patented ultrasound transit-time technology. The left donor lung was transplanted by three anastomoses in the following order: (1) bronchus with a running 4-0 PDS suture on the posterior and anterior walls; (2) LA cuff with a running 5-0 prolene suture; and (3) PA with a running 5-0 prolene suture as previously described. [11] After opening clamps, the graft was reperfused and the animal was monitored for 6 h. Whenever necessary, norepinephrine (Levophed, Pfizer Inc., US) was administrated intravenously for vasopressor support to maintain mean ABP above 50 mmHg starting with an initial dose of 8–12 mcg/min continuously. Lactate ringer was added (8 ml/kg/h) to maintain fluid balance. During implantation of the left lung, tidal volume (TV) was corrected due to right-single lung ventilation. To reflect this in our model, lungs were ventilated with a TV of 8 ml/kg and PEEP of 5 cmH_2_O during the baseline procedure and TV was reduced to 2/3 after pneumonectomy and during implantation. TV was then switched back to 8 ml/kg upon reperfusion. At the end of the experiment, animals were killed while on deep anesthesia by aortic clamping.

### Sampling

Upon reperfusion and during the monitoring period, blood samples were taken hourly from the carotid artery, PA via the indwelling catheter, and right and left pulmonary veins (RPV, LPV) by repeated direct puncture to monitor gas exchange.

**Fig. 1:**
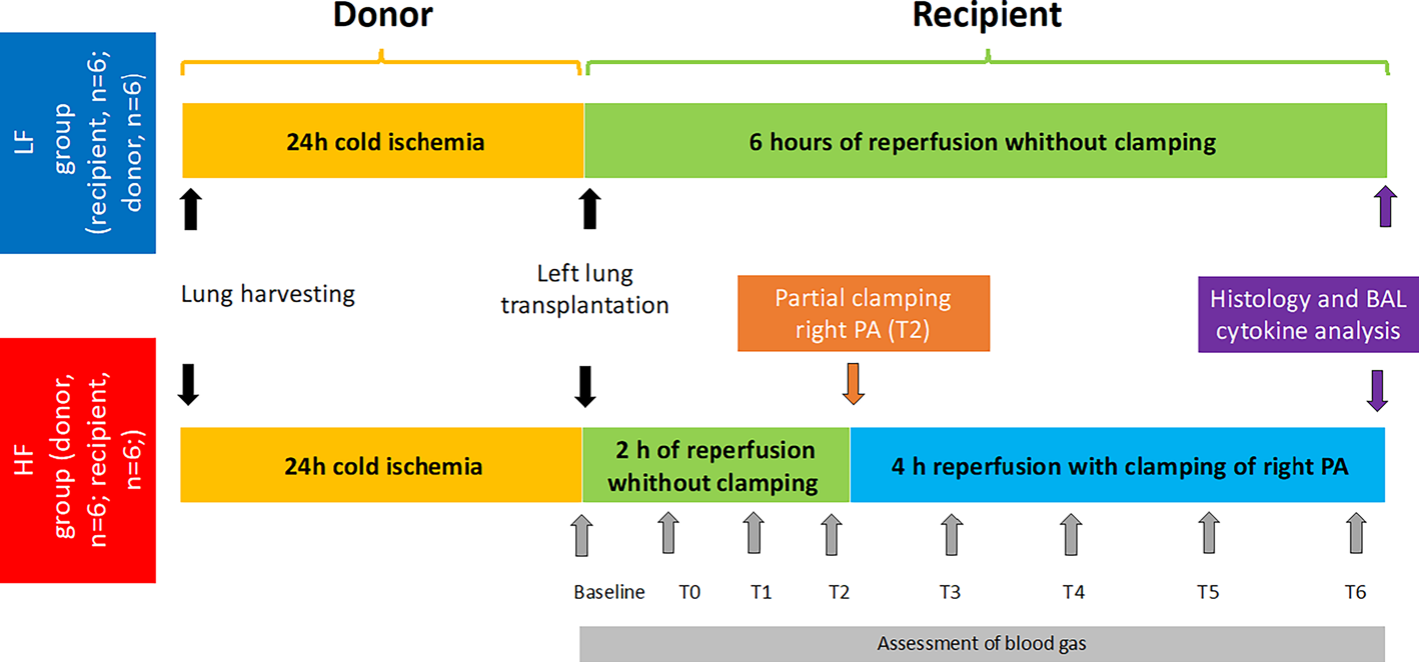
Schematic picture of experiment set-up. The figure demonstrates the set-up of the experiment of both groups. In both groups, a donor lung was harvested and stored for 24 h on ice. In a second animal (recipient), via left thoracotomy a left pneumonectomy was performed. In the low-flow (LF) group the reperfusion was observed for 6 h without partially clamping the right pulmonary artery. In the high-flow group (HF) the right pulmonary artery was partially clamped after the first 2 h of reperfusion for another 4 h

Differential blood gases from RPV and LPV allowed to discriminate the oxygenation capacity of the right native versus the left transplanted lung. In between sampling, the left chest cavity was closed temporarily and reopened hourly for sampling blood gases directly from the left and right pulmonary vein to measure differential oxygenation by both lungs while ventilated with FiO2 1.0 and PEEP 5 cm H_2_O. At the end of the experiment, a broncho-alveolar lavage (BAL) with two times 20 cc saline 0.9% was performed in the left lower lobe and the supernatant was analyzed with a porcine multiplex enzyme-linked immuno-sandwich assays (ELISA) kit for measurement of interleukin-6 (IL-6) and interleukin-8 (IL‐8) levels according to the manufacturer's protocol (R&D Systems, Inc. Minneapolis, MN, USA) with lower limits of quantification (LLOQ): 4.69 pg/ml for IL-6 and 31.25 pg/mg for IL-8 (Fig. 1).

Porcine multiplex ELISA according to the manufacturer's protocol (ThermoFisher, Scientific, Vienna, Austria) were performed on plasma samples, collected from each animal at baseline and at the end of the experiment for cytokine analysis, including interferon-α (IFN-α), interferon-γ (IFN-γ), interleukin-1beta (IL-1β), interleukin-10 (IL-10), interleukin-12p40 (IL-12p40), interleukin-4 (IL-4), interleukin-6 (IL-6), interleukin-8 (IL-8), and tumor necrosis factor-α (TNF-α) with lower limits of quantification (LLOQ): 0.6 pg/ml for IFN-α, 4.5 pg/ml for IFN-γ, 3.2 pg/ml for IL-1β, 18 pg/ml for IL-10, 30 pg/ml for IL-12p40, 1.5 pg/ml for IL-4, 5.9 pg/ml for IL-6, 16 pg/ml for IL-8, 6.5 pg/ml for TNF-α.

Lung biopsies were taken from the right and left lower lobe (RLL, LLL) from the recipient at the end of the experiment and from RLL of the (unused) donor lung at the end of the experiment and subsequently formalin fixed, paraffin embedded, and hematoxylin–eosin stained. Biopsies were scored for presence of interstitial widening, capillary congestion, intra-alveolar edema, hemorrhage, neutrophils in septa and in alveoli, and eosinophils in septa by a pathologist blinded for experimental groups. Also, biopsies for wet‐to‐dry weight (W/D) ratio calculation (after 72 h in the oven at 80 °C) were taken from the right and left lower lobe (RLL, LLL) to quantify lung edema [15].

### Statistical analysis

All data are described as median with interquartile range (IQR) (25% QI–75% QI) in GraphPad Prism 8 (GraphPad Software Inc, La Jolla, CA, USA). Values were compared between time points (T0–T6) and between both study groups using 2-way ANOVA for repeated measures or Mann–Whitney U-test and post hoc multiple comparison test Sidak (x). *p*-values of ≤ 0.05 was considered significant.

## Results

### Functional assessment during 6 h of reperfusion

Table 1 demonstrates parameters assessed at the time of baseline (before performing the left thoracotomy and left pneumonectomy in the recipient animal), at the time of reperfusion (T0), after 1 (T1), 2 (T2), 3 (T3), 4 (T4), 5 (T5) and 6 (T6) hours of reperfusion (Table 1).

**Table 1:** Outcome parameters from baseline till the end of reperfusion (T6)

Number of pigs, n	**Low flow**	**High flow**
	**6**	**6**
Donor		
Body weight, kg	50.5 (± 1.2)	49.5 (± 1.4)
pO2, mmHg	539.5 (450–568.3)	525 (452.5–555)
pCO2	41.7 (38.3–42.9)	42.5 (41.7–43.4)
Recipient		
Body weight, kg	52.7 (± 0.9)	52.3 (± 2.1)
Baseline		
mPAP, mmHg	24 (18.8–29.5)	22.5 (19–28.5)
pCO2	49.8 (44.4–52.1)	48.5 (39.6–50.8)
pO2 (LPV), mmHg	369 (287.8–531)	346 (308–417.5)
pO2 (RPV), mmHg	426.5 (395.5–453.5)	380 (321–407.5)
CO, L/min	3.85 (3.6–4.1)	4.7 (3.9–5.9)
Flow left PA, L/min	1.3 (0.9–1.8)	1.4 (0.9–1.9)
Reperfusion T0		
mPAP, mmHg	29.5 (19–36.3)	28.5 (22.8–30.3)
pCO2	47.4 (37.2–54.4)	46.3 (43.3–57.4)
pO2 (LPV), mmHg	300 (87.1–448)	315.5 (89.68–389.8)
pO2 (RPV), mmHg	325.5 (280.5–425.5)	371.5 (305.9–433.5)
CO, L/min	3.8 (3.3–5)	4.5 (3.8–6.4)
Flow left PA, L/min	0.18 (0.10–0.36)	0.17 (0.4–0.60)
Reperfusion T1		
mPAP, mmHg	27.5 (20.3–30.8)	27.5 (16.8–34.5)
pCO2	46.2 (42.6–52.3)	48.3 (44.9–54.2)
pO2 (LPV), mmHg	38 (319–441.8)	414.5 (256.5–452.5)
pO2 (RPV), mmHg	371 (297.3–460)	416 (344.3–448.3)
CO, L/min	38 (2.9–5.4)	4 (3.4–4.6)
Flow left PA, L/min	0.32 (0.19–0.48)	0.39 (0.25–0.95)
Reperfusion T2		
mPAP, mmHg	32.5 (28–34.5)	37 (29.3–42.3)
pCO2	43.6 (39.4–55.3)	47.9 (42.9–50.9)
pO2 (LPV), mmHg	352 (278.3–446.5)	291 (71.1–493)
pO2 (RPV), mmHg	404 (319.3–447.8)	410 (298.8–472.8)
CO, L/min	4.2 (3.3–4.7)	3.8 (3.1–4.2)
Flow left PA, L/min	1.1 (0.5–1.9)	1.5 (0.6–2.2)
Reperfusion T3		
mPAP, mmHg	28.5 (25.8–41.5)	38 (28.5–42.3)
pCO2	46.8 (43.7–49.1)	48.3 (44.6–54.5)
pO2 (LPV), mmHg	275 (194.9–358)	118.2 (79.3–296.5)
pO2 (RPV), mmHg	434 (392.5–477.5)	413.5 (316–451)
CO, L/min	3.7 (2.8–4.2)	4 (3.9–4.5)
Flow left PA, L/min	0.53 (0.43–0.66)	1.8 (1.1–2.2)
Reperfusion T4		
mPAP, mmHg	32 (21.5–40.3)	38.5 (34.8–45.3)
pCO2	46.2 (44.5–55.9)	48.3 (46.1–51.4)
pO2 (LPV), mmHg	270 (86.2–331)	97 (77.2–215.3)
pO2 (RPV), mmHg	397.5 (349.5–454.8)	322.5 (172.8–430.8)
CO, L/min	3.6 (3.3–4.4)	4.1 (3.4–4.9)
Flow left PA, L/min	0.69 (0.36–0.91)	1.7 (1.2–2.5)
Reperfusion T5		
mPAP, mmHg	27.5 (23.5–32)	42.5 (29.5–46.3)
pCO2	42.4 (37.3–46.3)	47.8 (43.6–53.6)
pO2 (LPV), mmHg	300.5 (193.8–355)	185.5 (70.3–234.3)
pO2 (RPV), mmHg	389.5 (351.8–445.8)	435.5 (369.8–486.5)
CO, L/min	3.5 (3–5.1)	4.2 (3.4–4.8)
Flow left PA, L/min	0.43 (0.36–0.59)	2.2 (1.1–2.5)

Data are expressed as median (25%–75% interquartile range); and Mann–Whitney was used for comparing the two groups

*CO* cardiac output, *PA *pulmonary artery, *pO*_*2*_ partial pressure of oxygen, *pCO*_*2*_ partial pressure of carbon dioxide, *LPV* left pulmonary vein, *RPV* right pulmonary vein, *mPAP* mean pulmonary arterial pressure, *T0* start of reperfusion (baseline), *T1* after 1 h reperfusion, *T2* after 2 h reperfusion, *T3* after 3 h reperfusion, *T4* after 4 h reperfusion, *T5* after 5 h reperfusion, *T6* after 6 h reperfusion, *W/D* wet-to-dry weight ratio, *RLL* right lower lobe, *LLL* left lower lobe, *LF* low flow, *HF* high flow

**Fig. 2:**
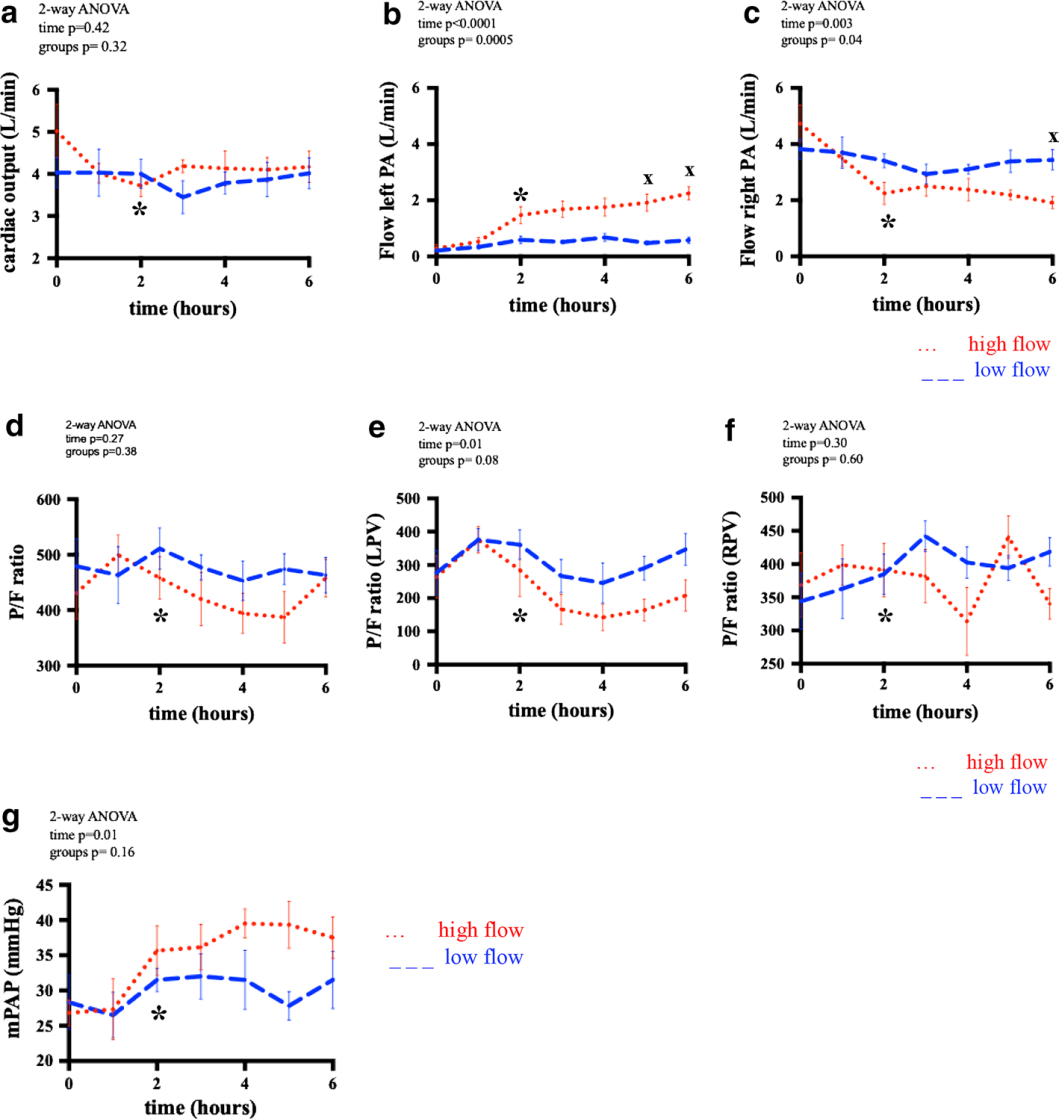
Parameters during reperfusion. **a**–**c** Assessment of hemodynamic parameters during 6-h reperfusion. CO and flow to the left and right lung were measured, flow through the right PA was calculated. All data are depicted as median ± IQR analyzed with repeated measures two-way ANOVA (**a**–**c**) and post hoc multiple comparison test Sidak (x). Time is 6-h reperfusion; CO, cardiac output; PA pulmonary artery; after 2 h of reperfusion, the right pulmonary artery was clamped in the high-flow group (*). **d**–**f** Assessment of oxygenation; blood gases samples were taken from carotid artery (P/F ratio), left pulmonary vein (LPV) and right pulmonary vein (RPV). All data are depicted as median ± IQR analyzed with repeated measures two-way ANOVA (**d**-**f**) and post hoc multiple comparison test Sidak (x). Time is 6-h reperfusion; pO2, partial pressure of oxygen; after 2 h of reperfusion, the right pulmonary artery was clamped in the high-flow group (*). **g** Assessment of pulmonary arterial pressure; all data are depicted as median ± IQR analyzed with repeated measures two-way ANOVA (G) and post hoc multiple comparison test Sidak (x). Time is 6-h reperfusion; mPAP, mean pulmonary arterial pressure; after 2 h of reperfusion, the right pulmonary artery was clamped in the high-flow group (*)

Physiological parameters assessed over the 6-h reperfusion period and are presented in Fig. 2.

Cardiac output was comparable over time between both groups (*p* = 0.32) (Fig. 2a).

As intended by the experimental design, minute blood flow to the left allograft over the 6 h was higher in HF (1.41 L) compared to LF (0.49 L); *p* = 0.0005 (Fig. 2b). Other way around, blood flow to the right native lung was significantly lower in HF (2.77 L vs 3.40 L; *p* = 0.04). Post hoc analysis demonstrated significant differences in flow to the allograft at 5 and 6 h (*p* = 0.03 and *p* = 0.0002, respectively) and in the native lung at 6 h (*p* = 0.04) of reperfusion (Fig. 2c).

P/F ratios of LPV and RPV were not significant (*p* = 0.08 and *p* = 0.60) (Fig. 2d–f).

**Fig. 3:**
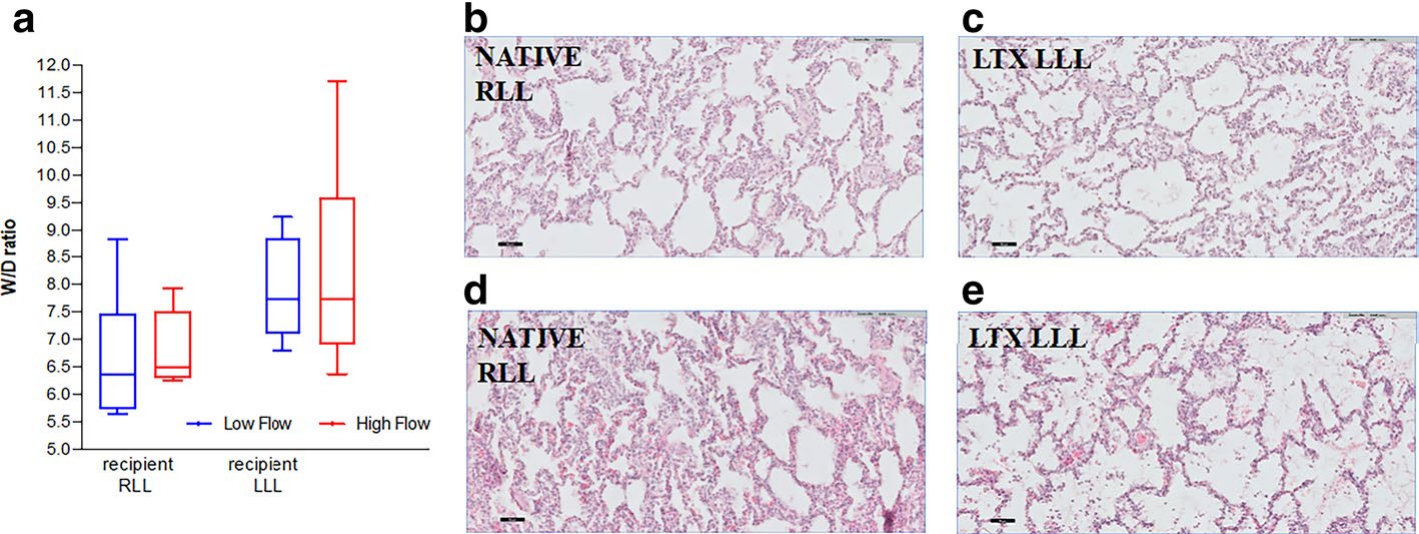
Histology. **a** The W/D ratios were assessed of lung biopsies at the end after 6 h reperfusion. No significant difference was observed between the low- vs. high-flow group in the right native lug (*p* = 0.49) and the left transplanted lung (*p* < 0.99). Data were analyzed with Mann–Whitney test; W/D, wet-to-dry weight ratio; RLL, right lower lobe; LLL, left lower lobe. *Left* (**b**) The native right lower lobe (RLL) of the low-flow (LF) group shows mild capillary congestion and mild septal neutrophilic infiltration without presence of intra-alveolar neutrophils. *Right* (**c**) The transplanted left lower lobe (LLL) of the low-flow (LF) group shows mild capillary congestion, presence of septal neutrophilic infiltration, intra-alveolar edema and intra-alveolar neutrophils. Left (**d**) The native right lower lobe (RLL) of the high-flow (HF) group shows prominent capillary congestion and presence of neutrophilic infiltration in the septa*. Right* (**e**) The transplanted left lower lobe (LLL) of the (HF) group shows presence of capillary congestion, prominent intra-alveolar edema and presence of septal and intra-alveolar neutrophilic infiltration

Mean PAP was not different in the HF group vs. the LF group (34.6 mmHg vs. 29.8 mmHg) (*p* = 0.16) (Fig. 2g). After 6 h reperfusion W/D of right native lung (*p* = 0.49) and left transplanted lung were similar (*p* > 0.99) (Fig. 3a).

### Immunological evaluation

Porcine multiplex ELISA analysis of the plasma at the end of the experiment between LF and HF group for the cytokines IFN-α, IFN-γ, IL-1β, IL-10, IL-12p40, IL-4, IL-6, IL-8 and TNF-α (*p* = 0.32) did not show any differences between both groups (Table 2). Similarly, no significant differences were demonstrated in the single cytokine ELISA analysis of BAL samples between the LF and HF group (IL-6, *p* = 0.23, IL-8, *p* = 0.07).

**Table 2:** Cytokine measurements in plasma of low- vs high-flow group

**Cytokines**	LLOQ (pg/ml)	LF	HF	*p*-value
IFN-alpha	0.6	0.5 (0.3–3.6)	0.9 (0.6–1.4)	0.33
IFN-gamma	4.5	5.4 (4.6–12.9)	13.9 (10.4–46.9)	0.10
IL-1beta	3.2	11.3 (5–299.1)	17.4 (8.3–32.9)	0.59
IL-10	18	56 (24.7–535.5)	73.5 (55.2 -89.2)	0.70
IL-12p40	30	510.1 (221.6–720.3)	265.5 (214.6–901.6)	0.82
IL-4	1.5	2.5 (0.7–5.4)	1.9 (1.8–2.5)	> 0.99
IL-6	5.9	73.5 (36.9–745)	150.4 (102.1–260.2)	0.18
IL-8	16	43.1 (22.1–1172)	30.4 (23.2–35.4)	0.67
TNF-alpha	6.5	101.7 (3.2–1139)	3.2 (3.2–85.7)	0.32

Cytokine measurements for the cytokines: interferon-α (IFN-α), interferon-γ (IFN-γ), interleukin-1β (IL-1β), interleukin-10 (IL-10), interleukin-12p40 (IL-12p40), interleukin-4 (IL-4), interleukin-6 (IL-6), interleukin-8 (IL-8) and tumor necrosis factor-α (TNF-α) were not significant in the low vs. high-flow group. Data are expressed as median (25%–75% interquartile range); and Mann–Whitney test was used for comparing the two groups; *LLOQ* lower limit of quantification, pg/ml, picogram/milliliter, *LF* low flow, *HF* high flow

### Histology

Histologic abnormalities in the left allograft and the right native lung were comparable between LF and HF groups (Fig. 3b–e).

Histological scoring of lung biopsies is shown in Table 3. In HF no differences were found between the right native lung and left allograft, though more neutrophils were observed in septa (*p* = 0.02) and neutrophils in the alveoli in the allograft compared to the native lung (*p* = 0.01).

**Table 3:** Lung biopsies from RLL and LLL in LF and HF group

**Group**	**LF**	**HF**		**LF**	**HF**		**LF**			**HF**		
**Location**	**LLL**		***p-value***	**RLL**		***p-value***	**RLL**	**LLL**	***p-value***	**RLL**	**LLL**	***p-value***
Pathological features												
Interstitial widening, grade 0–3	1.5 (1–2.25)	1 (1–1.25)	0.42	1.5 (1–2.25)	1 (1–1.25)	0.42	1.5 (1–2.25)	1.5 (1–2.25)	> 0.99	1 (1–1.25)	1 (1–1.25)	> 0.99
Capillary congestion, grade 0–2	2 (2–2)	2 (1.75–2)	> 0.99	2 (1.75–2)	2 (0.75–2)	0.73	2 (2–2)	2 (1.75–2)	> 0.99	2 (0.75–2)	2 (1.75–2)	0.73
Intra-alveolar edema, grade 0–2	2 (0.75–2)	1 (0–1.25)	0.21	0 (0–1.25)	0 (0–0)	0.45	0 (0–1.25)	2 (0.75–2)	0.13	0 (0–0)	1 (0–1.25)	0.06
Hemorrhage, grade 0–2	1 (0–2)	1.5 (1–2)	0.45	2 (0.75–2)	1 (0.75–2)	0.54	2 (0.75–2)	1 (0–2)	0.47	1 (0.75–2)	1.5 (1–2)	0.68
Neutrophils in septa, grade 0–3	3 (2–3)	2 (2- 3)	0.57	1.5 (1–2)	1.5 (1–2)	> 0.99	1.5 (1–2)	3 (2–3)	*0.02*	1.5 (1–2)	2 (2–3)	0.08
Neutrophils intra-alveolar, grade 0–2	2 (1.75–2)	1.5 (0–2)	0.30	0.5 (0–1)	0 (0–1.25)	> 0.99	0.5 (0–1)	2 (1.75–2)	*0.01*	0 (0–1.25)	1.5 (0–2)	0.37

Biopsies were scored for presence of interstitial widening, capillary congestion, intra-alveolar edema, hemorrhage, neutrophils in septa, and neutrophils intra-alveolar by a pathologist blinded for experimental groups. Gradings were from 0 (considered as absent) to grade of severity (1–3); LF, low flow; HF, high flow; LLL, left lower lobe; RLL, right lower lobe; data are expressed as median (25%–75% interquartile range); and Mann–Whitney test was used for comparing the two groups

## Discussion

In this study, we have introduced a novel approach to study the impact of pulmonary flow as a contributor to develop ischemia–reperfusion injury after one-lung transplantation in a large animal model. The unique aspect of our model is multiple. First, we demonstrate the feasibility of selective manipulation of pulmonary flow to investigate ischemia–reperfusion injury. Secondly, our model represents a specific intra-operative phase during sequential bilateral lung transplantation where the pulmonary flow is forced through the newly transplanted first lung. Finally, our model offers the possibility to further investigate extracorporeal circuits that deviate the flow from the right ventricle to control ischemia–reperfusion injury and to support right ventricular function.

Many researchers have developed models of one-lung transplantation in large animals [8–11, 16]. These models transplant a single allograft lung into a recipient animal. In some of these models, the contralateral lung is left untouched and unmodified and is still being fully perfused and ventilated. Others have excluded the non-transplanted lung completely from the circulation by clamping the hilum.

Therefore, these models have some major limitations in studying the ischemia–reperfusion injury.

First, when pulmonary flow is completely forced through the newly transplanted lung by excluding the native contralateral lung in the recipient animal, the perfusion injury might be irreversible since the pulmonary flow is too large. In addition, many of these models describe the need for circulatory support (type ECMO) to overcome hemodynamic instability caused by right ventricular failure (due to increased afterload).

Second, in the event, where the native contralateral lung is not clamped and fully integrated in the perfusion, it is difficult to control the flow through the newly transplanted lung. It might occur that due to high PVR, there is almost no flow passing through the vasculature of the transplanted lung. In this way, the model will not reflect a translational situation to study ischemia–reperfusion and ischemia might even be ongoing.

In order to overcome these problems, we have introduced a very innovative approach to better control the reperfusion of a newly transplanted lung in a large animal model by partially clamping the flow to the native lung and directly measuring the flow towards both lungs.

To avoid acute right ventricular failure, the right PA was only partially clamped as the non-dilatable suture line of the PA anastomosis of the left transplanted lung may create a relative obstruction and therefore cause an increased right ventricular afterload. Our technique to perform a wider PA anastomosis in our porcine LTx model was previously described [11].

All animals survived the 6-h reperfusion time and the partial PA clamping did not result in right ventricular failure right heart failure [17].

The impact of PA *flow* and physiological changes in the graft after reperfusion and in the early postoperative period is still debated [18, 19]. However, compared to systemic organs, cessation of blood flow results in hypoxia, except in the lungs where adequate tissue oxygenation can be maintained through ventilation only [20]. The terms “mechanotransduction, mechanosensing, mechanosignaling” are referring to a signaling cascade sensed by the pulmonary endothelium when blood flow ceases [21]. Endothelial mechanotransduction by abrupt cessation of blood flow to understand the role of ischemia-mediated ROS in signaling has been studied by other groups [21–26]. Al-Mehdi and colleagues demonstrated in a rat model that a low perfusate flow rate can prevent activation of the loss of shear stress signaling cascade (mechanotransduction) [27].

Overall, in our model, ischemia–reperfusion injury measured by physiological, histological and immunological variables did not significantly differ between the HF and LF group [4, 28]. This might be explained due to the limited amount of graft injury in the donor lung. Despite a long cold ischemic interval, donor animals had no additional injury related to typical events in clinical donors such as brain death or aggressive management. Also, reperfusion time of the transplanted graft was limited to 6 h only.

General *inflammatory markers* such as IFN-α, IFN-γ, IL-1β, IL-10, IL-12p40, IL-4, IL-6, IL-8, TNF-α measured in the plasma at the beginning and in the end of the experiment, were increased in both groups, showing activation of the innate immune system, without differences between study groups. In this study, measurements of immunologic markers, reflecting lung injury, were measured at a very early time point. Hamilton et al. describe biomarkers associated with PGD within the first 72 h post-LTx. There is a clear peak of biomarkers reflecting lung injury between 8 and 24 h after LTx [29] Therefore, it is questionable how much lung injury can already be observed after 6-h reperfusion like in our porcine LTx model. Interestingly, not only the transplanted left lung showed histological injury, also the right native lung was damaged as reflected in the LF group as mild capillary congestion and mild septal neutrophilic infiltration without presence of intra-alveolar neutrophils. In the HF group, histological injury of the right native lung showed shows prominent capillary congestion and presence of neutrophilic infiltration in the septa*.*

The remaining question regarding this observation is whether injury of the right native lung was caused by ventilation, reperfusion injury, spillover of toxic agents from the left lung, or due to systemic stress response to the transplantation procedure. Probably all these mechanisms together apply. This should be further investigated.

A direct clinical implication of our model might be the question if extracorporeal technology should be installed during the transplant process to deviate a fraction of the flow away of the newly transplanted lung. Our data suggest implementing a right-to-left bypass circuit might be an important strategy during double-lung transplantation to protect the first allograft from high pulmonary flow and early onset of ischemia–reperfusion injury. In clinical practice, extracorporeal support with cardiopulmonary bypass or veno-arterial ECMO is already often used during lung implantation. Our data suggest that the reduction of the flow to the first implanted lung might be an important mechanism to explain the protective nature of ECMO in the development of PGD. Of course, clinical decision-making is often based on PA pressures and gas exchange, where high PAPs and low P/F ratios are guiding the initiation of ECMO. Finally, in the clinical setting veno-arterial ECMO might also be considered to avoid right ventricular failure in addition to supporting pulmonary function.

Practices regarding the use of these ECMO devices vary among transplant centers and no randomized data are available [30–33]. A left-single lung transplantation survival model with clamping of the right pulmonary artery was chosen because sequential bilateral lung transplantation in pigs is not possible because of anatomic differences with the presence of a separate tracheal bronchus to the right upper lobe and an accessory right lower lobe draining into the left inferior pulmonary vein. Our model allows the study of this concept in the future.

## Limitations

Our study serves as a preclinical model to study ischemia–reperfusion injury. A potential limitation of our study is that we developed a porcine single-left lung transplantation model. This is because bilateral LTx in pigs is extremely difficult due to its anatomical variables compared to humans. Another limitation of our study is the fact that the left chest was left open after transplantation. This was necessary for technical reasons and control of clamping the right PA. Therefore, the ventilation data (compliance) are not reliable and do not reflect the compliance of the whole respiratory system. Also, we did not add a double-lumen tube and lung separation was not possible because of anatomical reasons (additional right upper lobe branching directly from the trachea). This adds to the fact that ventilation data could not separate left or right lung. Given these limitations, we have not reported on ventilatory parameters.

In addition, complete right hilar clamping is not feasible in a left lung transplanted pig for a 6-h survival model because of the high incidence of acute right heart failure. We realize that the absolute number of animals in each group is relatively low, especially to perform reliable statistical comparison. Nevertheless, we present a reproducible model with low variability in both groups. The primary goal of our study was to indicate the shortcomings of existing models and to open new perspectives to study ischemia–reperfusion injury in the future.

## Conclusions

Porcine single-lung transplantation models remain demanding, but the setting is feasible.

In this model, we could demonstrate the feasibility of selectively studying the impact of pulmonary flow to the transplanted lung. In the studied large animal model, differential blood flow did not impact the development of pulmonary IRI at 6 h of reperfusion.

However, our findings might have an impact on future studies about intra-operative problems during bilateral sequential single-lung transplantation with extracorporeal life support.

## Data Availability

The authors confirm that the data supporting the findings of this study are available within the article.

## Abbreviations

ABP: Arterial blood pressure
BAL: Broncho-alveolar lavage
CO: Cardiac output
ELISA: Enzyme-linked immuno-sandwich assays
ECLS: Extracoporeal life support
ETCO_2_: End-tidal carbon dioxide levels
HF: High flow
IFN-α: Interferon-α
IFN-γ: Interferon-γ
IL-1 β: Interleukin-1beta
IL-4: Interleukin-4
IL-6: Interleukin-6
IL-8: Interleukin-8
IL-10: Interleukin-10
IL-12p40: Interleukin-12p40
IVC: Inferior vena cava
IRI: Ischemia–reperfusion injury
L: Liters
LA: Left atrium
LAP: Left atrial pressure
LF: Low flow
LLL: Left lower lobe
LLOQ: Lower limits of quantification
LPV: Left pulmonary vein
LTx: Lung transplantation
P/F ratio: P_a_O_2_/F_i_O_2_ ratio
PA: Pulmonary artery
PAP: Pulmonary artery pressure
PEEP: Positive end-expiratory pressure
PGD: Primary graft dysfunction
PH: Pulmonary hypertension
PVR: Pulmonary vascular resistance
RLL: Right lower lobe
RPV: Right pulmonary vein
RR: Respiratory rate
RV: Right ventricular
SVC: Superior vena cava
TNF-α: Tumor necrosis factor-α
TV: Tidal volume
W/D: Wet-to-dry ratio
